# Possible seasonal and diurnal modulation of *Gammarus pulex* (Crustacea, Amphipoda) drift by microsporidian parasites

**DOI:** 10.1038/s41598-023-36630-2

**Published:** 2023-06-10

**Authors:** Sebastian Prati, Julian Enß, Daniel S. Grabner, Annabell Huesken, Christian K. Feld, Annemie Doliwa, Bernd Sures

**Affiliations:** 1 Aquatic Ecology and Centre for Water and Environmental Research, University of Duisburg-Essen, Universitaetsstr. 5, 45141 Essen, Germany; 2 Research Center One Health Ruhr, Research Alliance Ruhr, University Duisburg-Essen, Essen, Germany

**Keywords:** Animal migration, Behavioural ecology, Freshwater ecology

## Abstract

In lotic freshwater ecosystems, the drift or downstream movement of animals (e.g., macroinvertebrates) constitutes a key dispersal pathway, thus shaping ecological and evolutionary patterns. There is evidence that macroinvertebrate drift may be modulated by parasites. However, most studies on parasite modulation of host drifting behavior have focused on acanthocephalans, whereas other parasites, such as microsporidians, have been largely neglected. This study provides new insight into possible seasonal and diurnal modulation of amphipod (Crustacea: Gammaridae) drift by microsporidian parasites. Three 72 h drift experiments were deployed in a German lowland stream in October 2021, April, and July 2022. The prevalence and composition of ten microsporidian parasites in *Gammarus pulex* clade E varied seasonally, diurnally, and between drifting and stationary specimens of *G.* *pulex*. Prevalence was generally higher in drifting amphipods than in stationary ones, mainly due to differences in host size. However, for two parasites, the prevalence in drift samples was highest during daytime suggesting changes in host phototaxis likely related to the parasite’s mode of transmission and site of infection. Alterations in drifting behavior may have important implications for *G. pulex* population dynamics and microsporidians’ dispersal. The underlying mechanisms are more complex than previously thought.

## Introduction

Dispersal is a pervading feature of most animals and has an important role in shaping ecological and evolutionary patterns in natural populations^1,2^. Dispersal, or the movement of individuals across space, entailing potential consequences for gene flow^2^, is influenced by many factors. Among them, intra- and inter-specific competition (e.g., for food, habitat, mating partners), predation, habitat loss, lack of resources, and environmental stochasticity (e.g., flood and storm events) seem to be the prevailing mechanisms driving dispersal^3,4^. However, dispersal also depends on the individual performance of an organism^5^. Any alteration of the individual performance, including that directly or indirectly induced by parasites, may influence the ability and proneness of an organism to disperse.

Parasites can modulate animal behavior indirectly through pathogenicity, defense response induction, and directly via host manipulation^6–9^. Parasite-induced changes might, for instance, affect mobility^10^, habitat selection^7^, foraging^11^, reproduction^12^, longevity^13^, and host morphology^14^. Hence, the trade-off between the costs and benefits of dispersal might be influenced by parasites. For instance, seabirds might disperse more to escape infested habitats^15^. Meanwhile, condition-dependent dispersal in large terrestrial herbivores reduces the dispersal propensity of hosts with higher parasite load^16^. Parasites might also manipulate their host to disperse more frequently, increasing the chance of contact with the next host and thereby enhancing the transmission rate^17^. In aquatic ecosystems, for example, uninfected amphipods prefer dark and shaded areas. In contrast, conspecifics infected with acanthocephalan cystacanths show reverse geotaxis or reverse phototaxis and are more likely to be found in open water^18^. Such behavioral changes in intermediate hosts may enhance the individuals’ vulnerability to predation and thus lead to higher transmission rates^6,19,20^. However, host manipulation might also work in the opposite direction, e.g., by limiting drift in mermithid-infected mayfly nymphs to avoid predation by fish, which would result in the death of both host and parasite^21^. Nevertheless, not all parasite-induced alterations in host phenotype are part of an adaptative manipulation strategy. Some alterations may result from non-adaptive pathological side effects of infection^22^.

Amphipods are keystone species in aquatic ecosystems and generally harbor a wide range of parasites, some of which might affect dispersal^18,23^. Amphipods can actively disperse upstream to avoid resource limitation and competition, often in single massive migration events during times of limited food availability and high population densities^24,25^. On the other hand, drift or downstream dispersal with the water current can be either active or passive and is comparatively more consistent over time^24,25^. Drift plays a fundamental role in the population dynamics of amphipods and enables them to escape unfavorable conditions due to competition for food or predation risk^26^. Drift occurs mainly at night, about one hour after sunset and just before dawn^6,27,28^. In temperate regions, drift is often positively correlated with temperature, being at its lowest during winter and increasing toward summer^29^. However, like free-living biota, parasite communities vary seasonally due to temporal changes in abiotic and biotic factors, thus influencing the ecological dynamics of host-parasite relationships^30,31^. The burden of parasites in amphipods may fluctuate heavily between seasons and either impair host locomotory activity, as seen for the microsporidians *Pleistophora mulleri* and *Cucumispora ornata,* or enhance it, as for *Cucumispora dikerogammari*^23,32,33^. Hence, the influence of parasites on locomotory activity and, consequently, on host drifting behavior may result in alterations of host population dynamics that can be assumed to have broad ecological implications^34^.

The vast majority of studies looking at parasite modulation of host drifting behavior have focused on acanthocephalans^6,35,36^, whereas the influence of other parasites, such as microsporidians, remains largely unexplored. Microsporidians are a successful group of eukaryotic obligate intracellular parasites with relatively simple life cycles that exploit horizontal, vertical, and mixed-mode transmission to infect many hosts^37,38^. Horizontal transmission occurs via spore ingestion, venereally, or by direct invasion. Their transmission is often favored by cannibalistic behaviors, which are of common occurrence in amphipods^39^. On the other hand, vertical transmission occurs when spores are passed intergenerationally via transovarial transmission^37^. Generally, horizontal transmission is linked to high virulence, while vertical transmission is associated with low or no virulence or increased host fitness^32,40^. To our knowledge, only one study addressed the role of amphipod-infecting microsporidians on the drift behavior of their host^41^. The authors found that *Gammarus duebeni celticus* that were infected with the vertically transmitted microsporidian *Pleistophora* sp. were less abundant in the drifting fraction of the population. The influence of microsporidians and the differential role of their transmission routes on the drifting behavior of amphipod hosts remain largely unknown.

The present study aims to provide new insight into possible seasonal and diurnal modulation of amphipod drift by microsporidian parasites. Therefore, three drift experiments took place in October 2021, April, and July 2022, in a German lowland stream, the Rotbach, located within a nature-protected area. Three hypotheses drove our experimental design. Firstly, seasonal variations in microsporidian composition and prevalence are driven by shifts in amphipod body size distribution resulting from natural changes in age composition structure within amphipod populations. Secondly, horizontally transmitted microsporidians will be more represented in drifting amphipods than vertically due to possibly differential pathogenic effects on the host. And thirdly, microsporidians might alter the phototaxis of amphipods, resulting in a higher portion of infected individuals drifting during the day than at night. The mode of transmission of parasites might also influence drift timing due to different pathogenicity and site of infection.

## Methods

### Sampling

**Figure 1:**
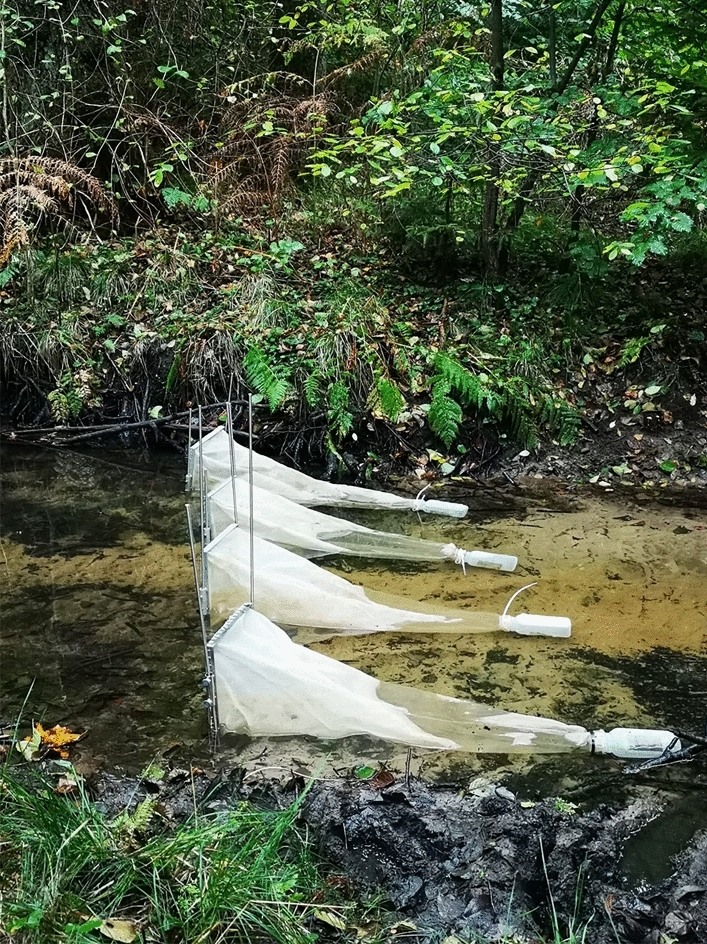
Drift nets placed diagonally in the stream to cover its width.

Amphipods were collected in three field experiments conducted in October 2021, April 2022, and July 2022. Each experiment lasted 72 h and took place in a nature-protected section of the Rotbach stream, North Rhine-Westphalia, Germany (51°34′03.4′′ N, 6°51′48.8′′ E). The Rotbach is a tributary of the Rhine, and its upper section, with only minimal anthropogenic disturbance, is one of the last standing natural sections of sand-bottom lowland streams in North Rhine-Westphalia and listed among the Federal State’s reference streams^42^. Drifting amphipods were collected at two hours intervals using drift nets (30 × 30 cm, mesh size 500 µm). The drift nets were placed diagonally to cover the entire width of the stream and avoid reciprocity bias in catches (Fig. 1). Water velocity was measured with an anemometer (Schiltknecht MC20 with C-53084 adapter) at 20% and 80% depth in the middle of each drift net at each emptying. Velocity varied depending on net placement, time, and season (range: 2–45 cm/s, mean ± SD: 20.1 ± 11.8 cm/s). After the conclusion of drift experiments, stationary amphipods were collected during the day, through kick sampling at two sites located 50 m and 100 m upstream of the uppermost drift net, in areas with differing flow velocity (range: 0.2–24 cm/s, mean ± SD: 12.8 ± 7.75 cm/s). The kick sampling consisted of triplicates for each flow velocity classes (low: < 7.5 cm/s, medium: 7.5–15 cm/s, and high > 15 cm/s) at each site.

All amphipods were immediately fixed in 96% ethanol. Amphipods were subsequently measured (fourth coxal plate length), morphologically identified to the lowest taxonomical level, dissected, and screened for parasites under the microscope. Moreover, amphipods were divided into four size classes based on coxal plate lengths (< 1, 1–1.99, 2–2.99, and > 3 mm) to investigate possible infection patterns related to ontogeny. Amphipod’s sex, however, was not initially recorded. In later samples, sex could not be assessed with certainty for most of the collected individuals, as distinctive sex characteristics are fully developed only in adults. Thus, sex was excluded from subsequent analyses. Only six individuals, one belonging to the stationary sample and five trapped in the drift nets, were infected with cystacanths of Acanthocephalans. Thus, acanthocephalans-infected amphipods were excluded from further analyses. Amphipods were dissected to investigate microsporidians, and guts removed to avoid detecting eventually enclosed spores. After gut removal, the remaining tissues were used for DNA extraction allowing the molecular identification of both host and microsporidian parasites. During a survey conducted between May and June 2021 at the same location, 40 amphipods were molecularly identified as belonging to *Gammarus pulex* clade E (99.8–100% similarity to KT075231). Therefore, an additional batch of 20 randomly selected individuals for each experiment was molecularly identified to account for possible variability in host composition between experiments for a total of 100 individuals.

If present, *G. pulex* eggs of infected specimens were extracted individually to identify possible vertical transmission of parasites. Additionally, to evaluate the potential role of fish in microsporidian spore dispersal, the gastrointestinal content of ten *Barbatula barbatula* individuals, the most common fish inhabiting the investigated stretch of the Rotbach, were also subject to DNA extraction. The fish were collected in the same area used for the drift experiment in May 2022 as part of an ongoing study of parasite diversity within the collaborative research center CRC 1439 RESIST framework.

### DNA isolation and sequencing

DNA was isolated from *G. pulex* tissues, individual eggs, and gastrointestinal fish content using a modified salt precipitation protocol according to Grabner et al*.*^43^. Molecular identification of hosts was obtained with the universal eukaryotic primers LCO1490 (5′-GGTCAACAAATCATAAAGATATTGG-3′) and HCO2198 (5′-TAAACTTCAGGGTGACCAAAAAATCA-3′)^44^, while that of microsporidians with the universal microsporidian-targeted primers V1 (5′-CACCAGGTTGATTCTGCCTGAC-3′)^45^ and mic-uni3R (5′-ATTACCGCGGMTGCTGGCAC-3′)^46^. PCR reaction volumes used for host tissues and gastrointestinal fish content were prepared following Weigand et al*.*^46^ using AccuStart II PCR ToughMix (Quanta Bioscience). One reaction contained 10 μL of 2 × ToughMix, 0.5 μM of each primer, and 1 μL of DNA. MilliQ water was added up to a total volume of 20 μL. Whereas PCR reaction volumes used for individual eggs were adjusted to account for a lower DNA yield than whole amphipods and gastrointestinal fish content by increasing the volume of DNA to 2 μL. PCR cycle conditions were set as follows: initial denaturation for 3 min at 94 °C, followed by 35 cycles of 35 s (host tissues) or 40 cycle of 35 s (eggs), denaturation at 94 °C and 40 s annealing at 68 °C, and a final elongation of 5 min at 68 °C. PCR products of hosts and microsporidians were sent to Microsynth Seqlab (Germany) for Sanger sequencing using LCO1490 and V1 primers, respectively.

### Sequences editing and alignment

Raw sequences were quality-checked and edited using Geneious v2023.0.1 (Biomatters). Only sequences with a minimum length of 200 bp were used for the analyses. Host and parasite sequences were separately aligned using the MAFFT v7.490 algorithm with standard settings^47^. Haplotypes of hosts and microsporidians were grouped in molecular operational taxonomic units (MOTUs) when the Kimura-2-parameter (K2p) corrected pairwise distances were below 2%. A threshold of 2% was chosen to account for potential intragenomic variation among microsporidians while remaining below commonly observed values of intraspecific variability in amphipods^43,48^. For microsporidians, a maximum likelihood phylogenetic tree with bootstrap support values (1000 replicates) was produced in IQ-Tree 2.2.0^49^. The TIM3 + F + G4 substitution model was selected based on Bayesian information criterion scores. To identify hosts and their microsporidian parasites, obtained MOTU sequences were compared against records contained in GenBank using megablast. The microsporidian *Metchnikovella dogieli* (MT969020) was used as an outgroup. The naming of undescribed Microsporidium isolates except for Microsporidium sp. RB 01, RB02, and RB03 followed the classification used by previous studies^50,51^.

### Statistical analyses

Statistical analyses were performed with the open-source software RStudio (version 2022.07.2, RStudio Inc.) based on R (version 4.2.2^52^).

Body size and flow velocity may influence drift, and parasitism, in turn, may influence both the body size and drift of amphipods^41^. However, the body size is expected to vary seasonally following the natural cycle of birth, growth, and death of *G. pulex*. Hence, the size (4th coxal plate) of pooled drifting and stationary amphipods was firstly compared between months using the Dunn post hoc test with FDR adjustment (rstatix package, version 0.7.1^53^) as data did not follow a normal distribution. Afterward, differences in *G. pulex* body size between stationary (day) and drifted samples (day and night) were compared for each part of the day and month separately, using a pairwise Wilcoxon rank sum test with FDR correction (rstatix package, version 0.7.1^53^).

A binomial generalized linear model (GLM) with parasite prevalence as the dependent variable and host body size, sample type (either stationary or drift), average flow velocity, and season as independent variables was employed to investigate the effect of body size on parasite prevalence in stationary and drifting amphipods. Since stationary amphipods were collected solely during the day, only the prevalence of amphipods drifting during the day was used in the model. The variance inflation factor (VIF; analyzed using the car package version 3.1-2^ 54^) remained below two for all descriptors. Multicollinearity was not an issue, and stepwise backward regression (MASS package version 7.3-56^55^) did not reduce the number of variables; thus, we retained all the variables. Including an interaction between sample type and season did improve the model. The results were reported as odd ratios. To assess diurnal differences in prevalence, we separated *G. pulex* in drift net samples into day (first and last sampling taken entirely in daylight) and night (sampling that included sunset, night, and sunrise). Prevalence differences in relation to diurnal drift were assessed with Fisher’s exact test (rstatix package, version 0.7.1^53^) for each experiment separately using pooled sample of parasites. When feasible, diurnal differences in prevalence were investigated for each parasite species separately using GLMs, and the results were reported as odd ratios. These were performed using parasite prevalence as the dependent variable, host body size, average flow velocity, and time of the day (either day or night) as the independent variable for each experiment separately.

## Results

**Figure 2:**
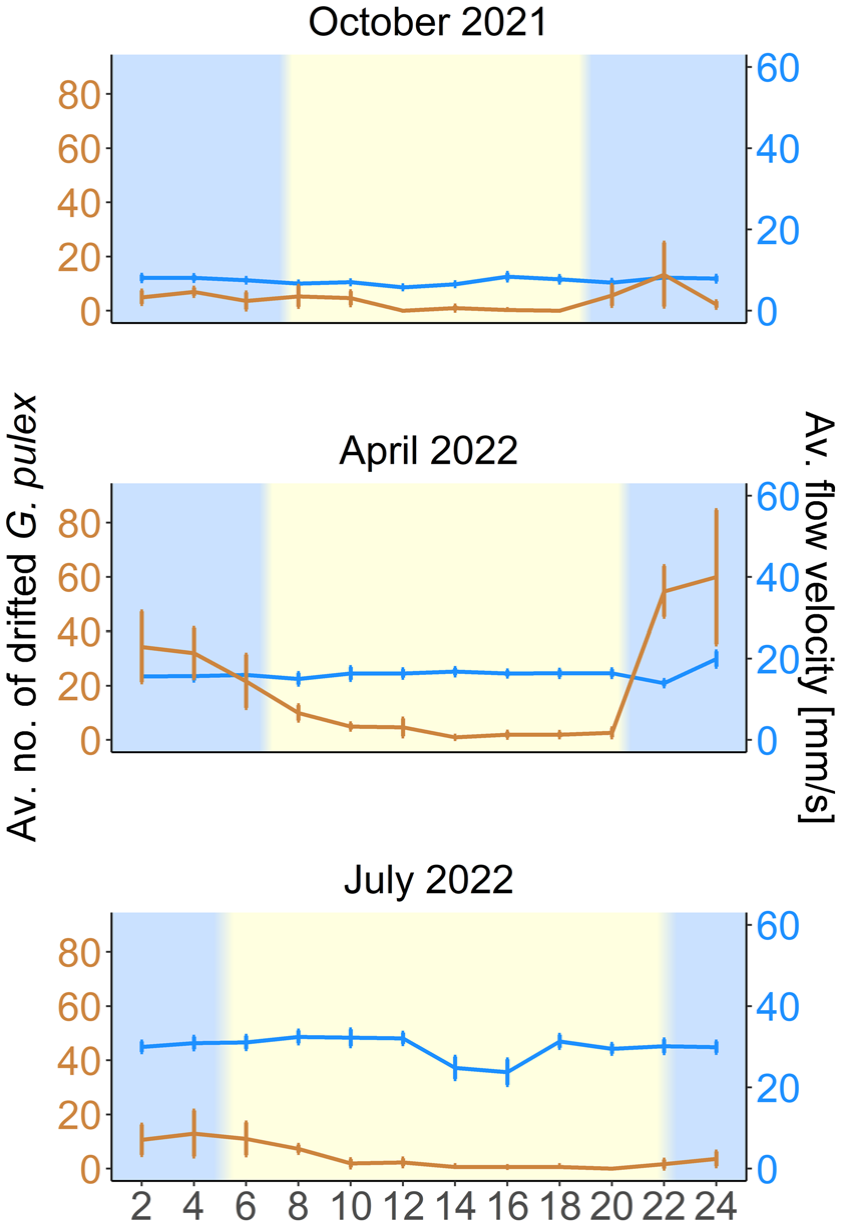
Line plot showing the average number of drifted *Gammarus pulex* captured in two hours intervals (in orange, each temporal point represents the average number of amphipods collected during the prior two hours) and the measured average water flow velocity in mm/s (in blue), including the standard error. The bluish background indicates night, while the yellowish background and transition areas represent day and sunrise/sunset. Please note that the two y-axes differ in scale.

The sample comprised 1893 *G. pulex* clade E specimens (all 100 molecularly identified individuals showed 99.7–100% similarity to KT075231). Of these, 897 individuals were collected by kick sampling, and 996 were trapped in drift nets. Drift rates of *G. pulex* peaked during the night before plunging during the daytime. This pattern remained stable across seasons irrespective of flow velocity (Fig. 2).

**Figure 3:**
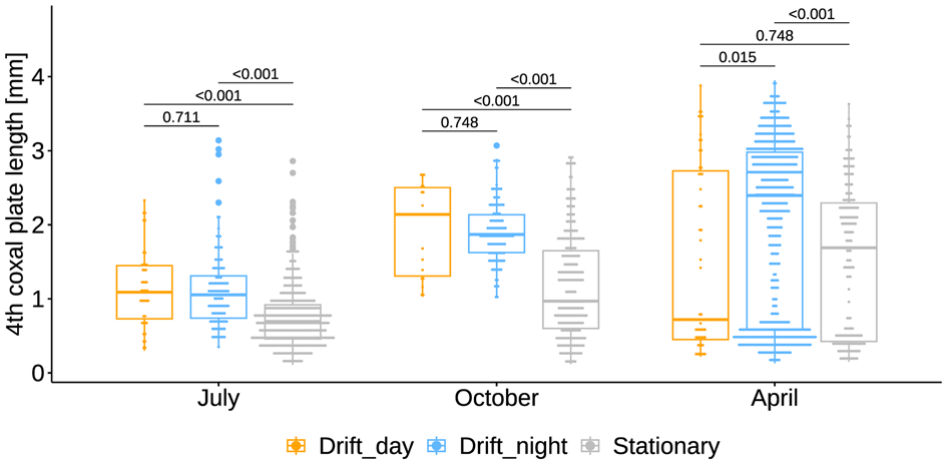
Size differences among drifted (day) and stationary (day and night) *Gammarus pulex* for each drift experiment. Horizontal bars within each experiment show size comparisons between drifted and stationary individuals, including *P*-values obtained pairwise Wilcoxon rank sum test with FDR correction.

*Gammarus pulex* body size differed substantially between seasons (Kruskal–Wallis test, H = 234, *d*ƒ = 2, *P* =  < 0.001), with larger individuals being more common in April and smaller individuals in July. However, in all three seasons, recently hatched *G. pulex* were detected, and in April, individuals were either large or very small (Fig. 3). On the other hand, their size distribution in October was more balanced. The observed differences were congruent for each season combination following pairwise comparisons (Dunn post hoc test, all *Padj* =  < 0.001). Moreover, the body size of drifted *G. pulex* was generally larger than that observed in stationary individuals (Wilcoxon rank sum test, all *P* =  < 0.001, Fig. 3).

**Figure 4:**
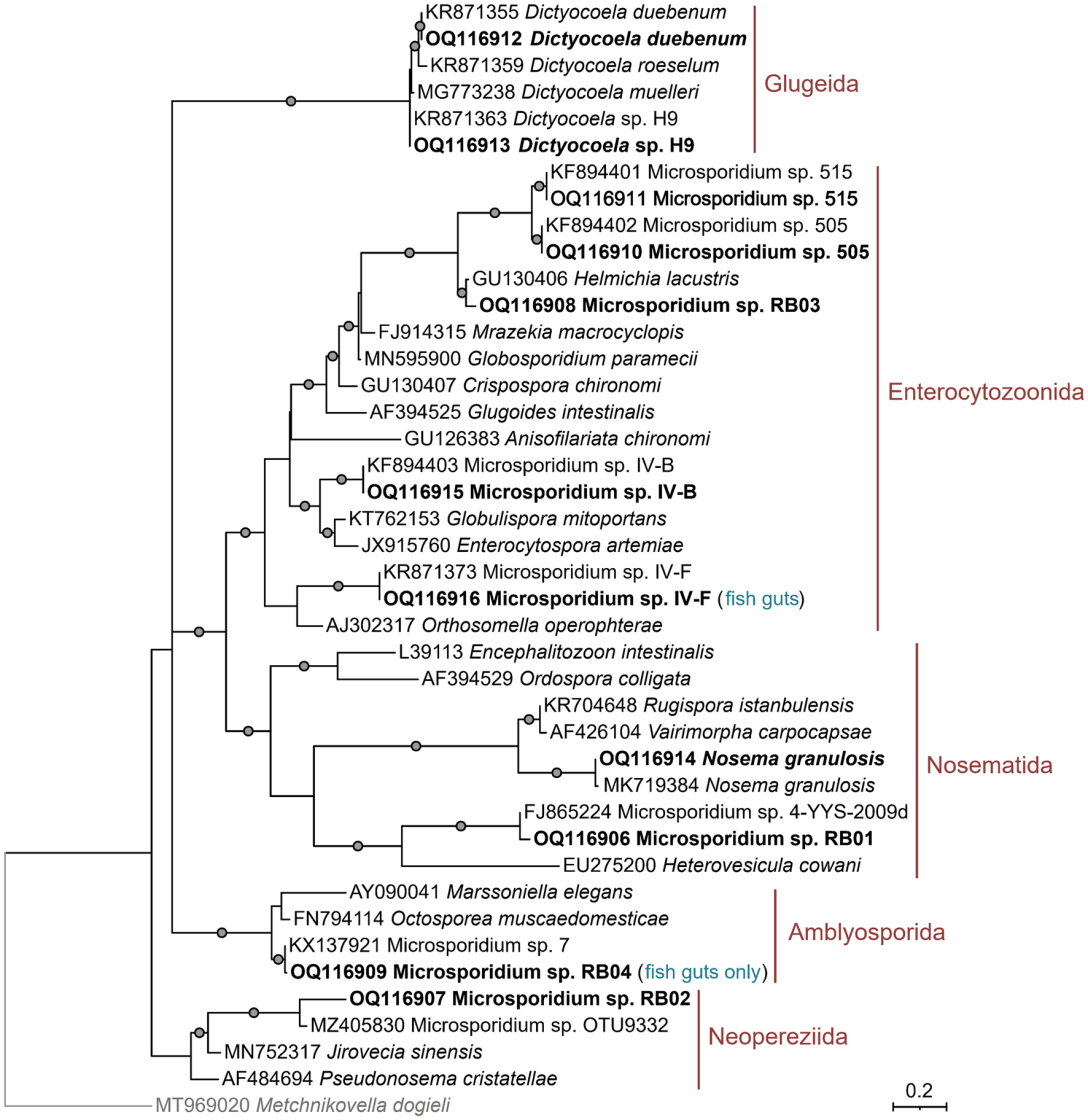
Maximum likelihood phylogenetic trees of microsporidians identified in amphipods and fish guts. The tree was obtained IQ-Tree 2.2.0 using the TIM3 + F + G4 substitution model. Dots represent bootstrap branch support values (1000 replicates) above 90%. The names and circumscriptions of microsporidians clades sensu Bojko et al*.*^56^ are indicated in red. Microsporidians, which were also present in fish guts or fish guts only, are noted in blue.

Ten microsporidian taxa belonging to four different clades sensu Bojko et al*.*^56^ were detected in *G. pulex* clade E (Fig. 4). Half of the identified microsporidians belonged to the Enterocytozoonida clade. These included Microsporidium sp. 505 (99.6–100% similarity to KX137937), Microsporidium sp. 515 (99.4–100% similarity to KX137939), Microsporidium sp. IV-B (100% similarity to KX137941), Microsporidium sp. IV-F (99.7–100% similarity to KR871373), and Microsporidium sp. 03RB. The latter is a possible new undescribed species showing 97.2% similarity to *Helmichia lacustris* (GU130406). Among them, Microsporidium sp. 505 and Microsporidium sp. 515 are supposedly horizontally transmitted, while Microsporidium sp. IV-B may be horizontally and vertically transmitted^57^.

Two microsporidians, *D. duebenum* (100% similarity to KR871355) and *Dictyocoela* sp. H9 (100% similarity to KR871363) belonged to the Glugeida clade. While *Nosema granulosis* (99.7–100% similarity to FN434088) and Microsporidium sp. 01RB (96.6–98% similarity to FJ865224 Microsporidium sp. 4-YYS-2009d, closest described species *Heterovesicula cowani* with 86% similarity) belonged to the Nosematida clade. *Nosema granulosis* and *Dictyocoela* spp. are usually vertically transmitted^50,58,59^. The remaining Microsporidium sp. 02RB belonged to the Neopereziida clade and showed 96.2% similarity to Microsporidium sp. OTU9332 (MZ405830), with the closely described species being *Jirovecia sinensis* (MN752318), with 86.3% similarity. Both Microsporidium sp. RB02 and RB03 were detected in single *G. pulex* individuals.

Among the 24 ovigerous females out of 61, a total of 126 eggs were molecularly screened for microsporidians. However, none of the eggs was infected. Molecular analyses of microsporidians in the fish gut revealed the presence of Microsporidium sp. IV-F (100% similarity to KR871373) and an unknown Microsporidium (99.3% similarity to KX137921 Microsporidium sp. 7, closest described species FN794114 *Octosporea muscaedomesticae* with 93.1% similarity) each in a single *B. barbatula* specimens.

Stationary *G. pulex* individuals were infected with all parasites except for Microsporidium sp. IV-F. Overall, the most prevalent microsporidian identified in this study was Microsporidium sp. IV-B, while Microsporidium sp. 515, and Microsporidium sp. 505 were the only microsporidians found every season and among drifted and stationary samples (Table 1). Microsporidian richness and prevalence were largely dependent on *G. pulex* size. The largest diversity of parasites was detected in the smallest specimens, while larger individuals had a higher prevalence than smaller ones (Table 2). Accordingly, the prevalence in *G. pulex* with a 4th coxal plate length above 2 mm ranged between 32.3 and 45.3%, while that in the two smaller size classes ranged between 4.18 and 9.59% (Table 2). Drifting *G. pulex* (day only) were generally larger than stationary individuals (4th coxal plate length range: 0.22–3.88 mm, mean ± SD: 1.45 ± 0.98 mm vs. range: 0.11–3.63 mm, mean ± SD: 1.04 ± 0.73 mm) and had a higher prevalence with 18% compared to 7.13%. Correspondingly larger specimens were more likely to be infected than smaller ones (OR 3.51, 95% CI 2.48–5.08), while stationary individuals were less likely to be infected than drifted ones (OR 0.36, 95% CI 0.14–0.94) primarily due to the smaller size of stationary *G. pulex* collected in July (OR 5.69, 95% CI 1.29–31.73). There was no evidence of flow velocity influencing parasite prevalence (OR 0.99 95% CI 0.96–1.03).

**Table 1:** Seasonal and diurnal prevalence of microsporidians in drifted and stationary (Station.) *Gammarus pulex* clade E for each drift experiment.

Microsporidian parasites	Microsporidian prevalence of drifted *Gammarus pulex* in %								
	October 2021			April 2022			July 2022		
	Drifted		Station.	Drifted		Station.	Drifted		Station.
	Day (n = 18)	Night (n = 127)	Day (n = 280)	Day (n = 52)	Night (n = 638)	Day (n = 187)	Day (n = 41)	Night (n = 120)	Day (n = 430)
*Dictyocoela duebenum*	0	0.79 (1)	0	0	0.47 (3)	0.5 (1)	0	0	0.2 (1)
*Dictyocoela* sp. H9	0	1.57 (2)	0.4 (1)	0	0	0.5 (1)	0	0	2.1 (9)
Microsporidium sp. 01RB	5.56 (1)	1.57 (2)	0	0	0	0	0	0	0.9 (4)
Microsporidium sp. 02RB	0	0	0	0	0	0	0	0	0.2 (1)
Microsporidium sp. 03RB	0	0	0	0	0	0	0	0	0.2 (1)
Microsporidium sp. 505	22.2 (4)	3.15 (4)	0.7 (2)	3.85 (2)	1.41 (9)	0.5 (1)	0	0.83 (1)	2.1 (9)
Microsporidium sp. 515	0	7.87 (10)	1.8 (5)	9.61 (5)	2.04 (13)	1.6 (3)	4.88 (2)	1.67 (2)	1.86 (8)
Microsporidium sp. IV-B	0	0	0	9.61 (5)	23.82 (152)	6.4 (12)	0	0	0.9 (4)
Microsporidium sp. IV-F	0	0	0	0	0.78 (5)	0	2.44 (1)	0.83 (1)	0
*Nosema granulosis*	0	0	0.4 (1)	0	0.16 (1)	0	0	0.83 (1)	0

The number of infected individuals is reported in brackets.

**Table 2:** Prevalence of microsporidians in *Gammarus pulex* clade E for each size class.

Microsporidian parasites	Microsporidian prevalence in *Gammarus pulex* for each size classes in %			
	< 1 mm (0.56 ± 0.22 mm, n = 885)	1–1.99 mm (1.49 ± 0.29 mm, n = 417)	2–2.99 mm (2.49 ± 0.29 mm, n = 412)	> 3 mm (3.29 ± 0.22 mm, n = 412)
*Dictyocoela duebenum*	0.23 (2)	0	0.24 (1)	0
*Dictyocoela* sp. H9	1.02 (9)	0.96 (4)	0.73 (3)	0
Microsporidium sp. 01RB	0.45 (4)	0.48 (2)	0.24 (1)	0
Microsporidium sp. 02RB	0.11 (1)	0	0	0
Microsporidium sp. 03RB	0.11 (1)	0	0	0
Microsporidium sp. 505	0.90 (8)	1.20 (5)	1.94 (8)	6.14 (11)
Microsporidium sp. 515	0.90 (8)	2.40 (10)	5.82 (24)	3.35 (6)
Microsporidium sp. IV-B	0.34 (3)	3.84 (16)	22.33 (92)	34.64 (62)
Microsporidium sp. IV-F	0.11 (1)	0.24 (1)	0.97 (4)	0.56 (1)
*Nosema granulosis*	0	0.48 (2)	0	0.56 (1)
Pooled samples	4.18 (37)	9.59 (40)	32.28 (133)	45.25 (81)

The number of infected individuals is reported in brackets.

Pooled microsporidians prevalence in drift (= 24 h) had minor variations between day and night in every season (Fisher’s exact test, all *p* > 0.184). However, when looking at each parasite separately, considering host size and average flow velocity, fewer *G. pulex* but with a higher prevalence of Microsporidium sp. 505 drifted during the day compared to the night in October and April samples (Table 1, Table S1). These differences were more evident in October than in April (OR 0.11, 95% CI 0.02–0.56 vs. 0.33, 95% CI 0.07–2.40). A similar pattern was observed in Microsporidium sp. 515 during April and July, with diurnal differences in prevalence more visible in the first (OR 0.13, 95% CI 0.04–0.43 vs. 0.32 95% CI 0.03–2.96). On the contrary, Microsporidium sp. IV-B, which was detected in the drift only in April, had a higher prevalence during the night. This, however, was mostly due to diurnal differences in host size (Table 1, Table S1). In none of the parasite taxa, flow velocity influenced prevalence (all OR 95% CI ranged from below 1 to above 1).

## Discussion

Seasonal parasite prevalence and composition variations driven by *G. pulex* size occurred in drifting and stationary samples. For two parasites, the prevalence in drifting *G. pulex* was highest during daytime, thus, suggesting changes in host phototaxis are likely related to the parasite’s mode of transmission or site of infection. Such findings underline the limited understanding of infection mechanisms related to microsporidians and their role in host population ecology. A better understanding of these mechanisms is crucial as any indirect and direct influence of parasites on host population structure and dynamics in keystone species such as amphipods are likely to affect ecosystem functioning^34,60^. Furthermore, the identification of three new isolates, Microsporidium sp. RB01, RB02, and RB03 in a single host population highlight the need for further research on this group of ubiquitous but often neglected parasites.

The present study expands the current knowledge of host-parasite interaction in *G. pulex* clade E by five interactions. Within the *G. pulex* complex, *Dictyocoela* sp. H09 was previously found in individuals belonging to the clade C, while *Nosema granulosis* in those of the clade D^43,61^. Microsporidium sp. RB01 is closely related to the isolate Microsporidium sp. 4-YS-2009d found in the barklouse *Polypsocus corruptus*^62^, and the closest described species is *H. cowani* which infects the adipose tissue of Mormon crickets, *Anabrus simplex*^63^. Microsporidium sp. RB02 is closely related to the isolate Microsporidium sp. OTU9332, for which no information relative to the host is available, as it was detected in lacustrine environmental samples^64^. However, the closest described species *J. sinensis* was recently described from a freshwater oligochaete, *Branchiura sowerbyi*^65^. Microsporidium sp. RB03, on the other hand, is closely related to *H. lacustris,* a microsporidian found in the midge larvae of *Chironomus plumosus*^66^. With half of the detected microsporidians representing new host-parasite interactions, the present finding highlights the need for further parasitological studies in amphipods.

Parasite composition varied between size classes, seasons, and sample types. Differences in host size mainly explained such variations. Overall, smaller individuals had lower parasite prevalence but a more variable community than the larger ones. This can happen if small differences in the exposure to parasites occurring at early stages generate large intraspecific differences, while the parasite community of larger individuals might become more homogeneous through repeated parasite exposure, as commonly observed in other aquatic organisms^67,68^. However, parasites appearing in early stages might become dominant over time, leading to the homogenization of parasite communities in larger individuals^69^. Homogenization of parasite communities in larger *G. pulex* individuals might also be favored by cannibalism and size-selective predation, the latter of which might be connected with the removal of large infected individuals (e.g., by predatory fish) and/or small infected individuals (e.g., by predatory macroinvertebrates such as dragonfly larvae) or by a combination of both^70,71^. Such mechanisms might, in turn, influence observed differences in parasite communities. Infections with several microsporidian taxa in one amphipod individual are considered rare as an initial infection may create a bottleneck for successive infections^72^. However, it was impossible to assess coinfections in the present study due to the limitation of the sequencing method used.

Like parasite composition, prevalence varied seasonally. Such differences were particularly evident in Microsporidium sp. IV-B. Interestingly, this species was not detected in October and was present in low prevalence during July but peaked in April. Seasonal differences in microsporidians prevalence were mainly explained by *G. pulex* size, with larger individuals being more infected than smaller ones. Accordingly, host size varied between seasons following natural population dynamics, with larger individuals mainly present in April, smaller ones in July, and middle-sized ones in October, supporting our first hypothesis. Such population dynamics are consistent with those observed in other *G. pulex* populations in which a cohort of juveniles was observed from spring throughout summer, while large individuals were nearly absent from July to October^73^.

A positive correlation between host size and parasite prevalence is commonly observed in aquatic organisms such as fish and snails^74,75^. As hosts grow, the exposure time to the parasite’s infective stages increases proportionally, enhancing the likelihood of becoming infected. Furthermore, ontogenetic dietary shifts leading to predation of conspecific and heterospecific individuals might occur, bolstering parasite infections. Size-asymmetric cannibalism is common in many taxa, including fish and aquatic invertebrates, mainly in size-structured species with overlapping generations in time and space^76^. Cannibalism is commonly observed in *G. pulex* and might potentially favor the transmission of parasites via ingestion of infected individuals^39^. In turn, microsporidian parasites might influence conspecific and heterospecific predatory interactions through enhanced aggression^77^.

Host size, likewise, explained differences in microsporidians prevalence between drifting and stationary *G. pulex*, with larger individuals being more common in the drift compared to stationary samples. This suggests that either large specimens are more dispersive or higher prevalence in larger specimens drives dispersal. Generally, a larger body size implies higher energy costs and food intake but also lowers locomotion costs per unit of body mass, favoring movement to richer patches and enhancing dispersal^78,79^. Accordingly, in a microcosm experiment, the home range of *G. insensibilis* was greater in larger individuals, as these could not fully exploit patches. On the other hand, smaller individuals with lower energetic requirements tended to use a limited set of patches^80^. Nevertheless, resources in the field might not be as limited as in experimental setups, and individual habitat preferences coupled with inter and intraspecific interactions might influence home range.

Although no clear pattern between *G. pulex* size and water flow was detected in the present study, smaller individuals are likely to prefer low water velocity near the banks^81^. In these microhabitats, leaves accumulate providing hiding opportunities from predation by larger conspecifics. Such habitat preferences may influence the size of *G. pulex* going into the drift. However, confounding mechanisms such as parasite pathogenicity might have a pivotal role. Accordingly, Microsporidium sp. 505, Microsporidium sp. 515, and Microsporidium sp. IV-B, all belonging to the Enterocytozoonida clade, were more often represented in drifting specimens than stationary ones, but the opposite was true for *D. duebenum* and *Dictyocoela* sp. H9, both belonging to the Glugeida clade. Similarly, in another study *G. duebeni celticus* infected with the microsporidian *Pleistophora* sp., belonging to the Glugeida clade, were less abundant in the drifting fraction of the population^41^.

Microsporidians of the Enterocytozoonida clade are generally tissue and organ-specific, mainly infecting epithelial cells of the midgut, particularly the hepatopancreas of aquatic arthropods. They can be transmitted horizontally and vertically or horizontally alone^56,82^. On the other hand, species belonging to the Glugeida clade are common in host muscle tissue and transmit horizontally, vertically, or both^56^. Correspondingly, vertical transmission is known in *D. duebenum* and other species of the same genus, which are found in the ovarian tissue and adjacent muscles^59,83^. Microsporidium sp. 505 and Microsporidium sp. 515 seem to be predominantly horizontally transmitted, while Microsporidium sp. IV-B is suspected of vertical transmission^57,59^. However, in the current study, no eggs of individuals infected with Microsporidium sp. IV-B tested positive, suggesting that horizontal transmission is the prevailing infection pathway in the studied area. The pristine status of the upper part of the Rotbach might possibly explain this discrepancy. Accordingly, in parasites with a mixed transmission mode, a switch from horizontal to vertical transmission may occur during phases of adverse environmental conditions and is an important survival strategy^84^. Alternatively, the PCR protocol used, although modified to maximize sensibility, might have failed to detect, if present, the very small number of spores contained in the eggs.

Horizontally transmitted parasites are generally linked to high virulence, which might reduce host fitness. For instance, *Enterocytozoon hepatopenaei,* a microsporidian belonging to the Enterocytozoonida clade, infects the hepatopancreas of various shrimps inducing storage consumption of lipids, downregulation of lipid metabolism and thus energy production^85^. It is, therefore, plausible that a reduction in fitness might occur and hinder the ability of *G. pulex* to withstand water flow and, thus, be more likely found in the drift. An enhanced drift of infected individuals is likely to benefit horizontally transmitted parasites by favoring spore dispersal. Although drift might result in spore dilution and limit cannibalism, many spores persist in the environment even in the absence of suitable hosts^86^. Depending on the microsporidian species, spores can be more or less resistant. Thus, from an evolutionary perspective, this might suggest that horizontally-transmitted parasites might produce more environmentally-resistant spores than vertically-transmitted parasites. If this was the case, long-lasting spores transported downstream might accumulate over time in areas of low water flow, reaching densities suitable for a successful infection. Amphipods likely use such areas as feeding grounds as leaves and other organic matter tend to accumulate. Infection may then occur when spores are inadvertently ingested during feeding activities.

Vertically-transmitted parasites are less virulent than horizontally-transmitted ones and can have either positive or neutral effects on host fitness^72^. They rely on successful host reproduction for their transmission. Hence host drifting might be counterproductive for several reasons. First, drift exposes the host to predation, possibly ending the parasite’s life cycle. Second, drift might reduce the host’s chances of encountering suitable partners due to a possible dilution effect. Amphipods are often aggregated and can have patchy distributions in streams^81,87^. Therefore, drifting from areas of higher to lower individual densities could impact *G. pulex* reproductive success hindering the vertical transmission of microsporidians. Differences in the site of infection, pathological stage, and host size may explain differences in the ratio of likely horizontally and vertically-transmitted microsporidians among drifting and stationary specimens.

Diurnal differences in the drift of *G. pulex* were observed only in individuals infected with Microsporidium sp. 505 and Microsporidium sp. 515 after accounting for size differences between day and night. Infected *G. pulex* seemed to drift mainly during daytime and to a lesser degree during nighttime. Such differences were particularly evident in October for Microsporidium sp. 505 and April for Microsporidium sp. 515. Drift activity of stream invertebrates typically is greatest during the nighttime hours in running waters throughout the world, presumably to minimize predation risk by visually hunting drift-feeding fishes^88^. Thus, the shift in the drift rate of infected individuals towards daytime suggests a change in the phototaxis of infected *G. pulex*, which supports the third hypothesis of our study.

Altered phototaxis has previously been observed in a variety of aquatic and terrestrial hosts infected by acanthocephalans, cestodes, fungi, nematomorphs, and trematodes. It is suspected to enhance transmission to the final host via predation or force the host to a suitable habitat where parasite life cycle completion can occur^89,90^. Only one experiment investigated phototaxis alteration in amphipods infected with the microsporidian *Dictyocoela roeselum.* This particular microsporidian did not have any relevant effect on phototaxis^91^. However, considering the contrasting life strategy of horizontally vs. vertically transmitted parasites, the opposite might be true for Microsporidium sp. 505 and Microsporidium sp. 515. An alteration of phototaxis toward daytime might be detrimental for vertically transmitted parasites as *G. pulex* might be more likely predated by visual feeding predators, hindering reproductive success and thus transmission. The same might not hold for horizontally transmitted parasites. A shift toward daytime might enhance predation risk, which could benefit the parasite by further enhancing spore dispersal following prey digestion. Their detection in fish guts suggests that these might be suitable vectors for amphipods infecting microsporidians. Even if there is no known phase of development in the fish, microsporidian spores might pass the digestive tract and be released into the environment while still being infective^92^. Therefore, microsporidians depending on their mode of transmission might benefit from the tendency of large amphipods to drift more, which may result in increased predation risk and consequently enhanced infections via spore dispersal even without direct manipulation of the host. Further studies, including morphological data, are thus required to shed light on the dispersal strategies of microsporidian parasites and the potential role of predators feeding on their hosts.

In conclusion, our findings show that microsporidian infections and population dynamics may influence host drift behavior. However, the underlying dispersal mechanism of microsporidian parasites with differing transmission routes might be more complex than previously thought, requiring further studies.

## Supplementary Information

Supplementary Information.

## Data Availability

The raw data supporting the conclusions of this article are available in the OSF repository, https://doi.org/10.17605/osf.io/b9tvk. The nucleotide sequence data reported are available in the GenBank database under the accession numbers OQ116906-OQ116916 for microsporidians and OQ121128 for *G. pulex* clade E.

## References

[1] Hansson L-A, Åkesson S (2014). Animal Movement Across Scales.

[2] Ronce O (2007). How does it feel to be like a rolling stone? Ten questions about dispersal evolution. Annu. Rev. Ecol. Evol. Syst..

[3] Bowler DE, Benton TG (2005). Causes and consequences of animal dispersal strategies: Relating individual behaviour to spatial dynamics. Biol. Rev..

[4] Gilliam JF, Fraser DF (2001). Movement in corridors: Enhancement by predation threat, disturbance, and habitat structure. Ecology.

[5] Clobert J, Le Galliard J-F, Cote J, Meylan S, Massot M (2009). Informed dispersal, heterogeneity in animal dispersal syndromes and the dynamics of spatially structured populations. Ecol. Lett..

[6] Lagrue C, Kaldonski N, Perrot-Minnot MJ, Motreuil S, Bollache L (2007). Modification of hosts’ behavior by a parasite: Field evidence for adaptive manipulation. Ecology.

[7] Żbikowska E, Cichy A (2012). Symptoms of behavioural anapyrexia—reverse fever as a defence response of snails to fluke invasion. J. Invertebr. Pathol..

[8] Moore J (2002). Parasites and the Behavior of Animals.

[9] Cézilly F, Thomas F, Médoc V, Perrot-Minnot M-J (2010). Host-manipulation by parasites with complex life cycles: Adaptive or not?. Trends Parasitol..

[10] Dezfuli BS, Maynard BJ, Wellnitz TA (2013). Activity levels and predator detection by amphipods infected with an acanthocephalan parasite, Pomphorhynchus laevis. Folia Parasitol..

[11] Fenton A, Rands SA (2006). The impact of parasite manipulation and predator foraging behavior on predator-prey communities. Ecology.

[12] Forbes MRL (1993). Parasitism and host reproductive effort. Oikos.

[13] Hurd H, Warr E, Polwart A (2001). A parasite that increases host lifespan. Proc. R. Soc. B.

[14] Miura O, Kuris AM, Torchin ME, Hechinger RF, Chiba S (2006). Parasites alter host phenotype and may create a new ecological niche for snail hosts. Proc. R. Soc. B.

[15] Brown CR, Brown MB (1992). Ectoparasitism as a cause of natal dispersal in cliff swallows. Ecology.

[16] Debeffe L (2014). Parasite abundance contributes to condition-dependent dispersal in a wild population of large herbivore. Oikos.

[17] Lion S, van Baalen M, Wilson WG (2006). The evolution of parasite manipulation of host dispersal. Proc. R. Soc. B..

[18] Sures B, Schmidt-Rhaesa A (2014). Ecology of the acanthocephala. Handbook of Zoology: Gastrotricha, Cycloneuralia and Gnathifera, Volume 3.

[19] Fayard M, Dechaume-Moncharmont F-X, Wattier R, Perrot-Minnot M-J (2020). Magnitude and direction of parasite-induced phenotypic alterations: A meta-analysis in acanthocephalans. Biol. Rev..

[20] Lafferty KD, Morris AK (1996). Altered behavior of parasitized killifish increases susceptibility to predation by bird final hosts. Ecology.

[21] Vance SA (1996). The effect of the mermithid parasite *Gasteromermis* sp. (Nematoda: Mermithidae) on the drift behaviour of its mayfly host, Baetis bicaudatus (Ephemeroptera: Baetidae): A trade-off between avoiding predators and locating food. Can. J. Zool..

[22] Williams JK, Townsend CR, Poulin R (2001). Mermithid nematode infections and drift in the mayfly *Deleatidium* spp. (Ephemeroptera). J. Parasitol..

[23] Bojko J, Ovcharenko M (2019). Pathogens and other symbionts of the Amphipoda: Taxonomic diversity and pathological significance. Dis. Aquat. Org..

[24] Žganec K, Gottsein S, Hudina S (2013). Spatio-temporal variation of drift and upstream movements of the Amphipod *Gammarus fossarum* in a small unaltered stream. Pol. J. Ecol..

[25] Goedmakers A, Pinkster S (1981). Population dynamics of three gammarid species (Crustacea, Amphipoda) in a French chalk stream. Part III. Migration. Bijdr. Dierkd..

[26] Brittain JE, Eikeland TJ (1988). Invertebrate drift—a review. Hydrobiologia.

[27] Elliott JM (2002). A continuous study of the total drift of freshwater shrimps, *Gammarus pulex*, in a small stony stream in the English Lake District. Freshw. Biol.

[28] Johnson JH (2014). Seasonal drift and feeding periodicity during summer of the amphipod, Gammarus psuedolimnaeus. J. Freshw. Ecol..

[29] Riel MCV, Der Velde GV, De Vaate AB (2011). Dispersal of invasive species by drifting. Curr. Zool..

[30] Holmes JC (1987). The structure of helminth communities. Int. J. Parasitol..

[31] Altizer S (2006). Seasonality and the dynamics of infectious diseases. Ecol. Lett..

[32] Fielding NJ (2005). Ecological impacts of the microsporidian parasite *Pleistophora mulleri* on its freshwater amphipod host *Gammarus duebeni celticus*. Parasitology.

[33] Bacela-Spychalska K, Rigaud T, Wattier RA (2014). A co-invasive microsporidian parasite that reduces the predatory behaviour of its host *Dikerogammarus villosus* (Crustacea, Amphipoda). Parasitology.

[34] Lefèvre T (2009). The ecological significance of manipulative parasites. Trends Ecol. Evol..

[35] McCahon CP, Maund SJ, Poulton MJ (1991). The effect of the acanthocephalan parasite (*Pomphorhynchus laevis*) on the drift of its intermediate host (*Gammarus pulex*). Freshw. Biol.

[36] Maynard BJ, Wellnitz TA, Zanini N, Wright WG, Dezfuli BS (1998). Parasite-altered behavior in a crustacean intermediate host: Field and laboratory studies. J. Parasitol..

[37] Dunn AM, Smith JE (2001). Microsporidian life cycles and diversity: The relationship between virulence and transmission. Microbes Infect..

[38] Wadi L, Reinke AW (2020). Evolution of microsporidia: An extremely successful group of eukaryotic intracellular parasites. PLoS Pathog.

[39] MacNeil C (2003). Parasite transmission and cannibalism in an amphipod (Crustacea). Int. J. Parasitol..

[40] Galbreath Slothouber JGM, Smith JE, Terry RS, Becnel JJ, Dunn AM (2004). Invasion success of Fibrillanosema crangonycis, n.sp., n.g: A novel vertically transmitted microsporidian parasite from the invasive amphipod host *Crangonyx pseudogracilis*. Int. J. Parasitol..

[41] MacNeil C, Dick JTA, Hatcher MJ, Dunn AM (2003). Differential drift and parasitism in invading and native *Gammarus* spp. (Crustacea: Amphipoda). Ecography.

[42] LANUV. *Leitbilder für kleine bis mittelgrosse Fließgewässer in Nordrhein-Westfalen*. https://www.lanuv.nrw.de/fileadmin/lanuvpubl/0_lua/merkbl17_webklein.pdf (1999).

[43] Grabner DS (2015). Invaders, natives and their enemies: Distribution patterns of amphipods and their microsporidian parasites in the Ruhr Metropolis, Germany. Parasit. Vectors.

[44] Folmer O, Black M, Hoeh W, Lutz R, Vrijenhoek R (1994). DNA primers for amplification of mitochondrial cytochrome c oxidase subunit I from diverse metazoan invertebrates. Mol. Mar. Biol. Biotechnol..

[45] Zhu X (1993). Small subunit rRNA sequence of *Enterocytozoon bieneusi* and its potential diagnostic role with use of the polymerase chain reaction. J. Infect. Dis..

[46] Weigand AM, Kremers J, Grabner DS (2016). Shared microsporidian profiles between an obligate (*Niphargus*) and facultative subterranean amphipod population (*Gammarus*) at sympatry provide indications for underground transmission pathways. Limnologica.

[47] Katoh K, Rozewicki J, Yamada KD (2019). MAFFT online service: Multiple sequence alignment, interactive sequence choice and visualization. Brief. Bioinform..

[48] Ironside JE (2013). Diversity and recombination of dispersed ribosomal DNA and protein coding genes in Microsporidia. PLoS ONE.

[49] Minh BQ (2020). IQ-TREE 2: New Models and Efficient Methods for Phylogenetic Inference in the Genomic Era. Mol. Biol. Evol..

[50] Bacela-Spychalska K (2018). Europe-wide reassessment of *Dictyocoela* (Microsporidia) infecting native and invasive amphipods (Crustacea): Molecular versus ultrastructural traits. Sci. Rep..

[51] Quiles A (2019). Microsporidian infections in the species complex *Gammarus roeselii* (Amphipoda) over its geographical range: Evidence for both host–parasite co-diversification and recent host shifts. Parasit. Vectors.

[52] R Core Team. R: A language and environment for statistical computing. R Foundation for Statistical Computing, Vienna, Austria (2021).

[53] Kassambara, A. rstatix: Pipe-Friendly Framework for Basic Statistical Tests. R package version 0.7.1. https://github.com/kassambara/rstatix/ (2023).

[54] Fox, J. *et al.* car: Companion to Applied Regression. R package version version 3.1-2. https://cran.r-project.org/package=car (2023).

[55] Ripley, B. *et al.* MASS: Support Functions and Datasets for Venables and Ripley’s MASS. R package version 7.3-56. https://cran.r-project.org/package=MASS (2023).

[56] Bojko J (2022). Microsporidia: A new taxonomic, evolutionary, and ecological synthesis. Trends Parasitol..

[57] Grabner DS, Schertzinger G, Sures B (2014). Effect of multiple microsporidian infections and temperature stress on the heat shock protein 70 (hsp70) response of the amphipod *Gammarus pulex*. Parasit. Vectors.

[58] Terry RS (1999). Ultrastructural characterisation and molecular taxonomic identification of Nosema granulosis n.sp., a Transovarially Transmitted Feminising (TTF) Microsporidium. J. Eukaryot. Microbiol..

[59] Terry RS (2004). Widespread vertical transmission and associated host sex–ratio distortion within the eukaryotic phylum Microspora. Proc. R. Soc. Lond. B Biol. Sci..

[60] Giari L, Fano EA, Castaldelli G, Grabner D, Sures B (2020). The ecological importance of amphipod–parasite associations for aquatic ecosystems. Water.

[61] Prati S, Grabner DS, Pfeifer SM, Lorenz AW, Sures B (2022). Generalist parasites persist in degraded environments: A lesson learned from microsporidian diversity in amphipods. Parasitology.

[62] Sokolova YY, Sokolov IM, Carlton CE (2010). New microsporidia parasitizing bark lice (Insecta: Psocoptera). J. Invertebr. Pathol..

[63] Sokolova YY, Lange CE, Fuxa JR (2008). Phylogenetic relationships of *Heterovesicula cowani*, a microsporidian pathogen of Mormon crickets, *Anabrus simplex* (Orthoptera: Tettigoniidae), based on SSU rDNA-sequence analyses. J. Invertebr. Pathol..

[64] Dubuffet A, Chauvet M, Moné A, Debroas D, Lepère C (2021). A phylogenetic framework to investigate the microsporidian communities through metabarcoding and its application to lake ecosystems. Environ. Microbiol..

[65] Liu X, Wen M, Zhao Y, Li A, Zhang J (2020). Morphological and molecular characterization of a new freshwater microsporidium, *Jirovecia sinensis* sp. n. (Microsporidia) infecting the coelomocytes of *Branchiura sowerbyi* (Oligochaeta: Naididae) in China. J. Invertebr. Pathol.

[66] Tokarev YS, Voronin VN, Seliverstova EV, Grushetskaya TA, Issi IV (2012). Ultrastructure and molecular phylogenetics of *Helmichia lacustris*, a microsporidium with an uncoiled isofilar polar filament. Parasitol Res.

[67] Timi JT (2011). Fish trophic level and the similarity of non-specific larval parasite assemblages. Int. J. Parasitol..

[68] Timi JT, Lanfranchi AL (2013). Ontogenetic changes in heterogeneity of parasite communities of fish: Disentangling the relative role of compositional versus abundance variability. Parasitology.

[69] Pérez-Del Olmo A, Fernández M, Raga JA, Kostadinova A, Poulin R (2008). Halfway up the trophic chain: Development of parasite communities in the sparid fish. Boops Boops. Parasitol..

[70] Wellborn GA (1994). Size-biased predation and prey life histories: A comparative study of freshwater amphipod populations. Ecology.

[71] Macneil C, Dick JTA, Elwood RW (1999). The dynamics of predation on *Gammarus* spp. (Crustacea: Amphipoda). Biol. Rev..

[72] Haine ER (2004). Coexistence of three microsporidia parasites in populations of the freshwater amphipod *Gammarus roeseli*: Evidence for vertical transmission and positive effect on reproduction. Int. J. Parasitol..

[73] Duran M (2007). Life cycle of *Gammarus pulex* (L.) in the river Yeşilırmak. Turk. J. Zool..

[74] Poulin R (2000). Variation in the intraspecific relationship between fish length and intensity of parasitic infection: Biological and statistical causes. J. Fish Biol..

[75] Soldánová M, Selbach C, Sures B, Kostadinova A, Pérez-del-Olmo A (2010). Larval trematode communities in *Radix auricularia* and *Lymnaea stagnalis* in a reservoir system of the Ruhr River. Parasit. Vectors.

[76] McGrath KE, Peeters ETHM, Beijer JAJ, Scheffer M (2007). Habitat-mediated cannibalism and microhabitat restriction in the stream invertebrate *Gammarus pulex*. Hydrobiologia.

[77] Bunke M, Dick JTA, Hatcher MJ, Dunn AM (2019). Parasites influence cannibalistic and predatory interactions within and between native and invasive amphipods. Dis. Aquat. Org..

[78] Makarieva AM, Gorshkov VG, Li B-L (2004). Body size, energy consumption and allometric scaling: A new dimension in the diversity–stability debate. Ecol. Complex..

[79] Dial KP, Greene E, Irschick DJ (2008). Allometry of behavior. Trends Ecol. Evol..

[80] Cozzoli F (2022). The size dependency of foraging behaviour: An empirical test performed on aquatic amphipods. Oecologia.

[81] Elliott JM (2005). Day–night changes in the spatial distribution and habitat preferences of freshwater shrimps, *Gammarus pulex*, in a stony stream. Freshw. Biol..

[82] Stentiford GD, Dunn AM, Weiss LM, Becnel JJ (2014). Microsporidia in aquatic invertebrates. Microsporidia: Pathogens of Opportunity.

[83] Dunn AM, Hogg JC, Hatcher MJ (2006). Transmission and burden and the impact of temperature on two species of vertically transmitted microsporidia. Int. J. Parasitol..

[84] Ebert D (2013). The epidemiology and evolution of symbionts with mixed-mode transmission. Annu. Rev. Ecol. Evol. Syst.

[85] Wu Y (2022). Down-Regulation of lipid metabolism in the hepatopancreas of shrimp *Litopenaeus vannamei* upon light and heavy infection of *Enterocytozoon hepatopenaei*: A comparative proteomic study. Int. J. Mol. Sci..

[86] Vizoso DB, Lass S, Ebert D (2005). Different mechanisms of transmission of the microsporidium *Octosporea bayeri*: A cocktail of solutions for the problem of parasite permanence. Parasitology.

[87] Welton JS (1979). Life-history and production of the amphipod *Gammarus pulex* in a Dorset chalk stream. Freshw. Biol.

[88] Flecker AS (1992). Fish predation and the evolution of invertebrate drift periodicity: Evidence from neotropical streams. Ecology.

[89] Andriolli FS (2019). Do zombie ant fungi turn their hosts into light seekers?. Behav. Ecol..

[90] Obayashi N (2021). Enhanced polarotaxis can explain water-entry behaviour of mantids infected with nematomorph parasites. Curr. Biol..

[91] Haine ER, Boucansaud K, Rigaud T (2005). Conflict between parasites with different transmission strategies infecting an amphipod host. Proc. R. Soc. B..

[92] Undeen AH (1990). A proposed mechanism for the germination of microsporidian (Protozoa: Microspora) spores. J. Theor. Biol..

