# Supplementary material for: Possible seasonal and diurnal modulation of Gammarus pulex (Crustacea, Amphipoda) drift by microsporidian parasites
Source: Sci Rep. 2023 Jun 10;13:9474. doi: 10.1038/s41598-023-36630-2 (PMC10257654; doi:10.1038/s41598-023-36630-2)
Supplement: Supplementary file 1 — Supplementary Information. [file 41598_2023_36630_MOESM1_ESM.pdf]

Table S1 Logistic regression output table from GLMs relative to factors (day/night, average flow velocity, and amphipod size) influencing microsporidians prevalence in *Gammarus pulex* clade E for each drift experiment and pooled experiment samples. The table includes coefficients, standard errors of coefficients, Z values, P values, Odd Ratios, Odd Ratios 95% confidence intervals, and McFadden pseudo-R^2.

Experiment	Variable	Coef.	SE Coef.	Z	P	OR	95% CI	Mcfadden pseudo-R^2
				Microsporidium sp. 505				
October 2021	Night	-2.225	0.835	-2.665	0.008	0.108	0.019-0.562	
	Size	1.288	0.818	1.574	0.115	3.625	0.782-20.478	0.165
	Av. flow	0.081	0.175	0.465	0.642	1.085	0.775-1.556	
April 2021	Night	-1.183	0.862	-1.297	0.195	0.327	0.070-2.397	
	Size	2.390	0.762	3.139	0.002	10.916	3.068-58.191	0.221
	Av. flow	0.126	0.105	1.198	0.231	1.134	0.926-1.405	
Pooled samples	Night	-1.904	0.534	-3.562	<0.001	0.149	0.054-0.453	
	Size	1.293	0.332	3.894	<0.001	3.644	2.013-7.496	0.175
	Av. flow	-0.083	0.050	-1.677	0.094	0.920	0.828-1.006	
				Microsporidium sp. 515				
April 2021	Night	-2.080	0.586	-3.549	<0.001	0.125	0.041-0.426	
	Size	0.785	0.270	2.908	0.004	2.193	1.349-3.961	0.117
	Av. flow	-0.096	0.076	-1.262	0.207	0.909	0.780-1.051	
July 2022	Night	-1.156	1.079	-1.071	0.284	0.315	0.032-2.956	
	Size	1.514	0.775	1.953	0.051	4.546	0.926-22.462	0.126
	Av. flow	0.017	0.096	0.180	0.857	1.017	0.822-1.226	
Pooled samples	Night	-1.198	0.457	-2.623	0.009	0.302	0.129-0.792	
	Size	0.567	0.195	2.900	0.004	1.762	1.216-2.628	0.058
	Av. flow	-0.035	0.029	-1.211	0.226	0.965	0.908-1.019	
				Microsporidium sp. IV-B				
April 2021	Night	0.928	0.520	1.783	0.075	2.528	0.983-7.861	
	Size	1.112	0.124	8.992	<0.001	3.041	2.414-3.927	0.178
	Av. flow	0.019	0.033	0.576	0.565	1.019	0.956-1.087	
